# Clinical decision-making and documentation gaps associated with influenza antiviral treatment in hospitalized children

**DOI:** 10.1017/ash.2026.10791

**Published:** 2026-07-22

**Authors:** Christina B. Felsen, Erin Licherdell, Maria A. Gaitan, Ghinwa Dumyati, Brenda Tesini

**Affiliations:** 1 Center for Community Health and Prevention, University of Rochester Medical Center, Rochester, NY, USA; 2 Department of Medicine, Infectious Diseases Division, University of Rochester Medical Center, Rochester, NY, USA; 3 Department of Pediatrics, Infectious Diseases Division, University of Rochester Medical Center, Rochester, NY, USA

## Abstract

Influenza antivirals are recommended for all hospitalized children. During 2023–2024, only 42% of hospitalized children with influenza in 7 counties in Western NY were treated. Comorbidities, illness severity, and symptoms predicted treatment. Parental declination, emphasis of treatment risk, and providers’ application of outpatient guidelines to inpatients also played a role.

## Introduction

Influenza causes approximately 45,000 pediatric hospitalizations in the United States annually.^1^ Antiviral therapy can reduce disease severity, duration and complications^2^ and is recommended for all hospitalized children with influenza.^3^ The proportion of hospitalized children with influenza who received antiviral treatment decreased from 78% in 2017–2018 to 56% in 2023–2024.^4^ We evaluated antiviral prescribing in a regional cohort of children hospitalized with influenza to identify factors associated with treatment and documentation of treatment decisions in electronic health records (EHR).

## Methods

This analysis included children aged 0–17 years residing in 7 counties in Western NY, hospitalized at 2 hospitals between October 2023-April 2024 with laboratory-confirmed influenza during hospitalization or within the preceding 14 days. We abstracted demographic and clinical information from the EHR using standardized CDC FluSurv-NET methodology.^5^ Treated children received oseltamivir, baloxavir, peramivir, or zanamivir for any duration, during or prior to hospitalization. Severe disease was defined as intensive care unit admission or receipt of oxygen via high-flow nasal cannula, bilevel/continuous positive airway pressure, or invasive mechanical ventilation. To identify factors associated with antiviral use, we performed bivariate analysis and multivariable logistic regression to estimate odds ratios and 95% confidence intervals (Wald method) using SAS 9.4.

Two authors abstracted antiviral non-prescription reasons verbatim from the EHR. Following a thematic analysis process,^6^ 3 authors selected and reviewed relevant keywords from abstracted quotations to develop themes. The study was deemed exempt by the University Institutional Review Board.

## Results

### Patient characteristics, presentation and treatment

Among 131 children hospitalized with influenza during the 2023–2024 season, 55 (42%) received an antiviral, mainly oseltamivir (95%). Two children started treatment before hospitalization. Most children (84%) were hospitalized at the region’s academic children’s hospital; treatment did not differ significantly between hospitals. The median age and length of stay was 5 years (IQR 2–9) and 2 days (IQR 1–3), respectively. Sixty-four percent of children had an underlying condition, 24% were severely ill, and 2 died.

Most children (96%) had respiratory symptoms. Children treated with antivirals more often had shortness of breath (60% vs 39%), wheezing (42% vs 22%), and were hospitalized sooner after respiratory symptom onset (median: 3 d (IQR 2–4) vs 4 d (IQR 3–7)). Non-respiratory symptoms including fever (96%) and gastrointestinal (GI) symptoms (70%) were common. Sixty children (46%) received antibiotics. Few eligible children were vaccinated (25%), with higher rates among treated children (35%) (Table 1).

**Table 1.** Characteristics of hospitalized children stratified by receipt of antivirals, 2023–2024 Table 1 long description.

	Total	Not treated	Treated	
Characteristic	n (%)			*P*-value
No. of cases	131	76 (58.0)	55 (42.0)	
Median age, years (IQR)	5 (2–9)	5 (3–9)	5 (2–12)	0.48
Sex, Male	82 (62.6)	47 (61.8)	35 (63.6)	0.83
Race/Ethnicity				
Non-Hispanic White	65 (49.6)	38 (50.0)	27 (49.1)	
Non-Hispanic Black	36 (27.5)	19 (25.0)	17 (30.9)	
Non-Hispanic Asian	2 (1.5)	1 (1.3)	1 (1.8)	0.11
Unknown	11 (8.4)	4 (5.3)	7 (12.7)	
Hispanic, any race	17 (13.0)	14 (18.4)	3 (5.5)	0.03
≥ 1 Underlying condition	84 (64.1)	41 (54.0)	43 (78.2)	0.004
Underlying conditions				
Asthma/reactive airway disease	38 (29.0)	17 (22.4)	21 (38.2)	0.05
Other chronic lung disease	13 (9.9)	5 (6.6)	8 (14.6)	0.13
Chronic metabolic disease	7 (5.3)	4 (5.3)	3 (5.5)	1.00
Blood disorders/hemoglobinopathy	2 (1.5)	0	2 (3.6)	0.17
Cardiovascular disease	13 (9.9)	6 (7.9)	7 (12.7)	0.36
Neurologic disorders	45 (34.3)	23 (30.1)	22 (40.0)	0.25
Immunocompromised condition	11 (8.4)	7 (9.2)	4 (7.3)	0.76
Renal disease	3 (2.3)	1 (1.3)	2 (3.6)	0.57
Obesity	10 (7.6)	6 (7.9)	4 (7.3)	1.00
Gastrointestinal/liver disease	3 (2.3)	0	3 (5.5)	0.72
Hypertension	1 (0.8)	0	1 (1.8)	0.42
Mental health conditions	3 (2.3)	1 (1.3)	2 (3.6)	0.57
Prematurity^a^	13 (11.4)	5 (6.9)	8 (19.1)	0.07
Other pediatric conditions^a,b^	10 (8.8)	5 (6.9)	5 (11.9)	0.49
≥ 1 Documented respiratory symptom^c^	126 (96.2)	74 (97.4)	52 (94.6)	0.65
Respiratory symptoms^c^				
Chest congestion	9 (7.1)	5 (6.8)	4 (7.7)	1.00
Congested/runny nose	114 (90.5)	64 (86.5)	50 (96.2)	0.12
Cough	116 (92.1)	67 (90.5)	49 (94.2)	0.52
Shortness of breath/increased work of breathing	60 (47.6)	29 (39.2)	31 (59.6)	0.02
Sore Throat	36 (28.6)	22 (29.7)	14 (26.9)	0.73
Upper respiratory infection/Influenza like illness	48 (38.1)	28 (37.8)	20 (38.5)	0.94
Wheezing	38 (30.2)	16 (21.6)	22 (42.3)	0.01
Apnea^a^	2 (1.8)	1 (1.4)	1 (2.6)	1.00
Cyanosis^a^	4 (3.7)	2 (2.9)	2 (5.1)	0.62
Nasal flaring/grunting/retractions^a^	27 (24.8)	13 (18.6)	14 (35.9)	0.06
Stridor^a^	3 (2.8)	3 (4.3)	0	0.55
Gastrointestinal symptoms^c^				
Abdominal pain	37 (28.2)	25 (32.9)	12 (21.8)	0.18
Nausea/vomiting	66 (52.0)	44 (59.5)	22 (41.5)	0.05
Diarrhea	32 (24.2)	21 (27.6)	11 (20.0)	0.41
Median time between respiratory symptom onset and hospital admission, days (IQR)^d^	4 (2–5)	4 (3–7)	3 (2–4)	0.007
Markers of disease severity				
ICU admission	15 (11.4)	4 (5.3)	11 (20.0)	0.001
Non-invasive respiratory support^e^	20 (15.3)	5 (6.6)	15 (27.3)	0.001
Invasive mechanical ventilation	6 (4.6)	0	6 (10.9)	0.01
Antibiotics received during hospitalization	60 (45.8)	28 (36.8)	32 (58.2)	0.02
Vaccinated > 14 days before positive flu test^f^	29 (25.0)	11 (16.9)	18 (35.3)	0.04
Median length of stay, days (IQR)	2 (1–3)	1 (1–2)	2 (1–6)	0.01
In-hospital death	2 (1.5)	0	2 (3.6)	0.17
Antiviral agent given	–	–		
Oseltamivir			52 (94.5)	
Peramivir			3 (5.5)	

a Only collected for children < 12 years of age (n =114).

b Includes abnormality of airway, chronic lung disease of prematurity/bronchopulmonary dysplasia, and history of febrile seizures.

c Symptom present at the time of admission or developed/worsened in the 14 days prior.

d Among children with symptom onset before admission, documented respiratory symptoms, and known onset date (n = 117).

e Receipt of supplemental oxygen via bipap/cpap or high-flow nasal cannula.

f Limited to patients over 6 months of age with known vaccination status, defined as a full date of vaccination found in the EHR and/or the New York State Immunization Information System (n =116). Vaccinated defined as receipt of at least one dose >14 days before positive flu test and during the current respiratory viral season.

### Predictors of antiviral use

In unadjusted bivariate analysis, a hospital stay of 2 days or more (OR 3.0, 95% CI 1.4, 6.2), having at least 1 underlying condition (OR 3.1, 95% CI 1.4, 6.7), severe disease (OR 5.7, 95% CI 2.1, 15.5) and current vaccination for influenza (OR 2.7, 95% CI 1.1, 6.4) were associated with treatment. Children with GI symptoms (OR 0.4, 95% CI 0.2, 0.9) and those identifying as Hispanic (OR 0.3, 95% CI 0.1, 0.9) were less frequently treated. In adjusted models, severe disease (OR 4.6, 95% CI 1.6, 5.1) and the presence of at least 1 underlying condition (OR 2.4, 95% CI 1.1, 5.5) remained significant predictors of treatment (Table 2).

**Table 2.** Unadjusted and multivariable logistic regression of the relationship of clinical and demographic characteristics to antiviral prescription (n = 131) Table 2 long description.

Unadjusted results	
Exposure	Odds ratio (95% Confidence interval)
Age (years)	
<1	Ref
1–4	0.6 (0.2, 2.0)
5–11	0.4 (0.1, 1.4)
12–17	3.3 (0.7, 14.4)
Race	
White, NH	Ref
Black, NH	1.3 (0.6, 2.9)
Asian/unknown, NH	2.3 (0.7, 7.6)
Hispanic ethnicity	
No	Ref
Yes	0.3 (0.1, 0.9)
Length of stay (days)	
<2	Ref
≥2	3.0 (1.4, 6.2)
Respiratory symptoms^a^	
No	Ref
Yes	0.5 (0.1, 2.9)
GI Symptoms^a^	
No	Ref
Yes	0.4 (0.2, 0.9)
≥1 Underlying condition	
No	Ref
Yes	3.1 (1.4, 6.7)
Severe disease^b^	
No	Ref
Yes	5.7 (2.1, 15.5)
Time between respiratory symptom onset and admission (days)^c^	
<4	Ref
≥4	0.6 (0.3, 1.3)
Vaccinated (does not include unknown, only includes those with known status, n = 116)	
No	Ref
Yes	2.7 (1.1, 6.4)
**Multivariable results**	
≥ 1 GI symptom^a^, ^d^	
No	Ref
Yes	0.4 (0.2, 1.0)
≥ 1 Respiratory symptom^a^, ^e^	
No	Ref
Yes	1.5 (0.2, 12.9)
≥ 1 Underlying condition ^f^	
No	Ref
Yes	2.4 (1.1, 5.5)
Severe disease^g^	
No	Ref
Yes	4.6 (1.6, 5.1)
Vaccinated^h^	
No	Ref
Yes	2.4 (1.0, 5.8)

a Present at admission or developed/worsened in the 2 weeks prior to admission.

b Intensive care unit admission or receipt of oxygen via high-flow nasal cannula, bilevel/continuous positive airway pressure, or invasive mechanical ventilation.

c Includes only children with symptom onset before admission, documented respiratory symptoms, and known onset date (n = 117).

d Adjusted for length of stay, severe disease, underlying conditions, age group, race, and Hispanic ethnicity.

e Adjusted for Hispanic ethnicity, GI symptoms, race, and time between respiratory symptom onset and hospital admission.

f Adjusted for vaccination status.

g Adjusted for length of stay.

h Adjusted for age group and underlying conditions; limited to children eligible for vaccination with known vaccine status (n = 116).

### Documentation of antiviral non-prescription

Half of the 76 untreated children lacked a documented reason for antiviral non-prescription. Documentation for the remaining children (Supplemental Table 1) showed that antivirals were not given due to:

#### Parental declination

Some families declined antiviral treatment due to concern over “significant GI side effects” while others declined “to avoid side effects” after discussion with a provider.

#### Provider risk/benefit communication

Documentation of provider/family discussions often focused on potential side effects of oseltamivir, stating that the benefit was “not worth the risk.”

#### Treatment guideline adherence variation

Providers frequently documented symptom duration “greater than 48 hours” as a reason for non-prescription, noting that patients were “outside of the recommended treatment window.” Antivirals were also withheld among patients that were perceived to be less severely ill due to “primarily GI symptoms” or no “acute respiratory concerns.”

#### Patient medication intolerance

Poor oral medication tolerance was documented, specifically among children with GI symptoms. Occasionally, oseltamivir was prescribed but not administered due to refusal or inability to take oral medication; treatment was not reinstituted after GI symptom resolution.

### Discussion

In this study, only 42% of children hospitalized with laboratory-confirmed influenza received antivirals, consistent with post COVID-19 pandemic trends.^4,7^ Clinical presentation, illness severity, and underlying comorbidities influenced antiviral prescribing. Documentation in the EHR suggested the following additional factors impacted treatment: (1) parental declination, (2) provider emphasis on risks over benefits, (3) guideline adherence variation, and (4) oral medication tolerance concerns.

Gaps in clinical decision-making documentation were prevalent; half of untreated children lacked EHR documentation regarding antiviral non-prescription. A study evaluating oseltamivir use among infants in inpatient and outpatient settings found that 40% of untreated infants lacked non-prescription documentation.^8^ Poor documentation of antibiotic prescribing is also prevalent, highlighting the role of tools such as clinical decision-supports (eg, EHR smart forms) to prompt better recording of treatment decisions.^9^ Improved documentation could identify knowledge gaps or misconceptions to inform targeted influenza antiviral education.

Treated children were more likely to have comorbidities or severe illness, suggesting clinicians prioritize treatment for higher risk children. This aligns with prior studies showing that providers often rely on perceived illness severity, rather than guideline-based criteria alone, to influence treatment.^4,10,11^ Children admitted earlier in their illness were more likely to receive antivirals despite evidence that later antiviral initiation can still reduce risk of severe outcomes in hospitalized children.^12^ Providers often documented patients as “outside the [48 hour] window” for antiviral treatment, suggesting the application of outpatient treatment guidelines^3^ to hospitalized inpatients. In a recent national survey, one-third of clinicians were unaware of treatment recommendations and practices varied among those familiar with the guidelines, reflecting differing interpretations of the limited pediatric inpatient evidence.^11^ Decision-support tools that provide guideline rationale may improve adherence and address provider uncertainty.

GI manifestations are common in children with influenza.^2^ They were highly prevalent in our cohort and associated with lower antiviral use due to documented concerns for symptom exacerbation. Inpatient antimicrobial stewardship efforts could include clearer guidance on managing antiviral therapy when GI symptoms are present and oral intake is limited. Antibacterials were prescribed more often than antivirals, despite similar GI side effects and low bacterial co-infection rates, making this a high-yield stewardship opportunity.

Parental concerns about side effects contributed to antiviral non-prescription and were often noted following provider discussions of treatment risk. These findings underscore the importance of providers offering unbiased recommendations that outline both potential benefits and risks of treatment during shared decision-making.^13^

This study is subject to several limitations. Data are from a single influenza season and children hospitalized at 2 hospitals in Western NY, limiting generalizability. Influenza testing was at the discretion of the provider, and our data reflects only what was documented in the EHR, which may not capture the full clinical decision-making process. Despite these limitations, our findings identify patient, clinician, and family factors influencing antiviral treatment decisions among hospitalized children with influenza.

Antiviral prescribing in our cohort was low despite national recommendations for treatment of hospitalized children; treatment decisions were influenced by illness duration and severity. Documentation suggests that concerns about side effects and varied application of treatment guidelines also influenced prescribing. Better understanding of the drivers of these decisions may help inform strategies to improve guideline-concordant antiviral use.

## Supporting information

10.1017/ash.2026.10791.sm001 Felsen et al. supplementary material Felsen et al. supplementary material

## Data Availability

The de-identified data and code supporting the findings of this study are available from the corresponding author upon reasonable request.

## Supplementary material

The supplementary material for this article can be found at https://doi.org/10.1017/ash.2026.10791.

## Table 1. Long description

The table presents characteristics of hospitalized children stratified by receipt of antivirals, 2023-2024. It has 131 rows and 10 columns. The columns are labeled as Characteristic, Total, Not treated, Treated, and P-value. The rows are labeled with various characteristics such as No. of cases, Median age, Sex, Race/Ethnicity, Underlying conditions, Respiratory symptoms, Gastrointestinal symptoms, Markers of disease severity, and Antiviral agent given. Each row provides specific data points for each characteristic. For example, Row 1: No. of cases, 131; Not treated, 76; Treated, 55; P-value, empty. Row 2: Median age, years (IQR), 5 (2-9); Not treated, 5 (3-9); Treated, 5 (2-12); P-value, 0.48. The table captures detailed data on various characteristics and their distribution among treated and not treated groups.

Navigate back to Table 1..

## Table 2. Long description

A table with 18 rows and 3 columns showing unadjusted and multivariable logistic regression results. The columns are labeled Exposure, Odds ratio, and 95 percent Confidence interval. The table is divided into two sections: Unadjusted results and Multivariable results. Each row lists different exposures such as Age, Race, Hispanic ethnicity, Length of stay, Respiratory symptoms, GI Symptoms, Underlying condition, Severe disease, Time between respiratory symptom onset and admission, and Vaccinated. The values in the Odds ratio and 95 percent Confidence interval columns vary for each exposure. Notable trends include higher odds ratios for hospital stay greater than 2 days, having at least 1 underlying condition, severe disease, and current vaccination for influenza in unadjusted results. Children with GI symptoms and those identifying as Hispanic have lower odds ratios. In adjusted models, severe disease and the presence of at least 1 underlying condition remain significant predictors of treatment.

Navigate back to Table 2..
