# Supplementary material for: Clinical decision-making and documentation gaps associated with influenza antiviral treatment in hospitalized children
Source: Antimicrob Steward Healthc Epidemiol. 2026 Jul 22;6(1):e223. doi: 10.1017/ash.2026.10791 (PMC13419408; doi:10.1017/ash.2026.10791)
Supplement: Felsen et al. supplementary material [file S2732494X26107918sup001.docx]

Table 3. Themes and Illustrative Quotes: Reasons for Antiviral Non-Prescription

| **Themes^a^** | **Illustrative Quotes** |
| --- | --- |
| Parental declination of antivirals | “Mother reported that she did not wish to start this medication as there have been other family members on Tamiflu previously that had significant GI side effects”  “Tamiflu was discussed with his father, including benefits and side effects, father opted to refuse”  “Parents elected to hold off at this time given side effect risks and that patient is already improving” |
| Provider communication about the risk/benefit of antivirals | “We discussed possibility of using Tamiflu, but given that patient has had symptoms for >48 hours, family and patient opted not to use it to avoid side effects”  “Offered Tamiflu but explained side effects, mom declined”  “Prior to admission discussed with mom that she is slightly outside of the appropriate time range for Tamiflu also discussed possibility of GI side effects with mom, she endorsed understanding.” |
| Provider misunderstanding of treatment guidelines | “Although treating with oseltamivir could be considered, there would be no benefit given the absence of respiratory symptoms and negative pulmonary exam”  “Out of window for Tamiflu”  “Hold off Tamiflu, as no acute respiratory concerns, no immunocompromised status and mostly GI and poor PO symptoms”  “Outside of 48 hour window and symptoms not severe at this time, will not dose Tamiflu” |
| Patient medication intolerance | “Stopped Tamiflu given significant emesis”  “Hold off on Tamiflu in the setting of PO refusal”  “Hold on Tamiflu in the setting of GI distress” |

^a^ Themes were developed using a thematic analysis process. Two team members (E.L. and C.F.) abstracted the reasons for antiviral non-prescription verbatim from the EHR. Antiviral receipt was verified using medication administration records with additional context obtained from clinical notes. If no documented rational was found, targeted searches using terms such as “antiviral” and “Tamiflu” were performed. Three team members (E.L., C.F., and B.T.) independently reviewed all abstracted data to identify key words. These team members met to compare key words and group them into themes and then collaboratively assigned themes to all data. Agreement on conflicting interpretations was achieved via group discussion.
